# Supplementary material for: Sperm Functional Status: A Multiparametric Assessment of the Fertilizing Potential of Bovine Sperm
Source: Vet Sci. 2024 Dec 23;11(12):678. doi: 10.3390/vetsci11120678 (PMC11680172; doi:10.3390/vetsci11120678)
Supplement: Supplementary file 1 [file vetsci-11-00678-s001.zip › Supplemental Figure S1.pdf]

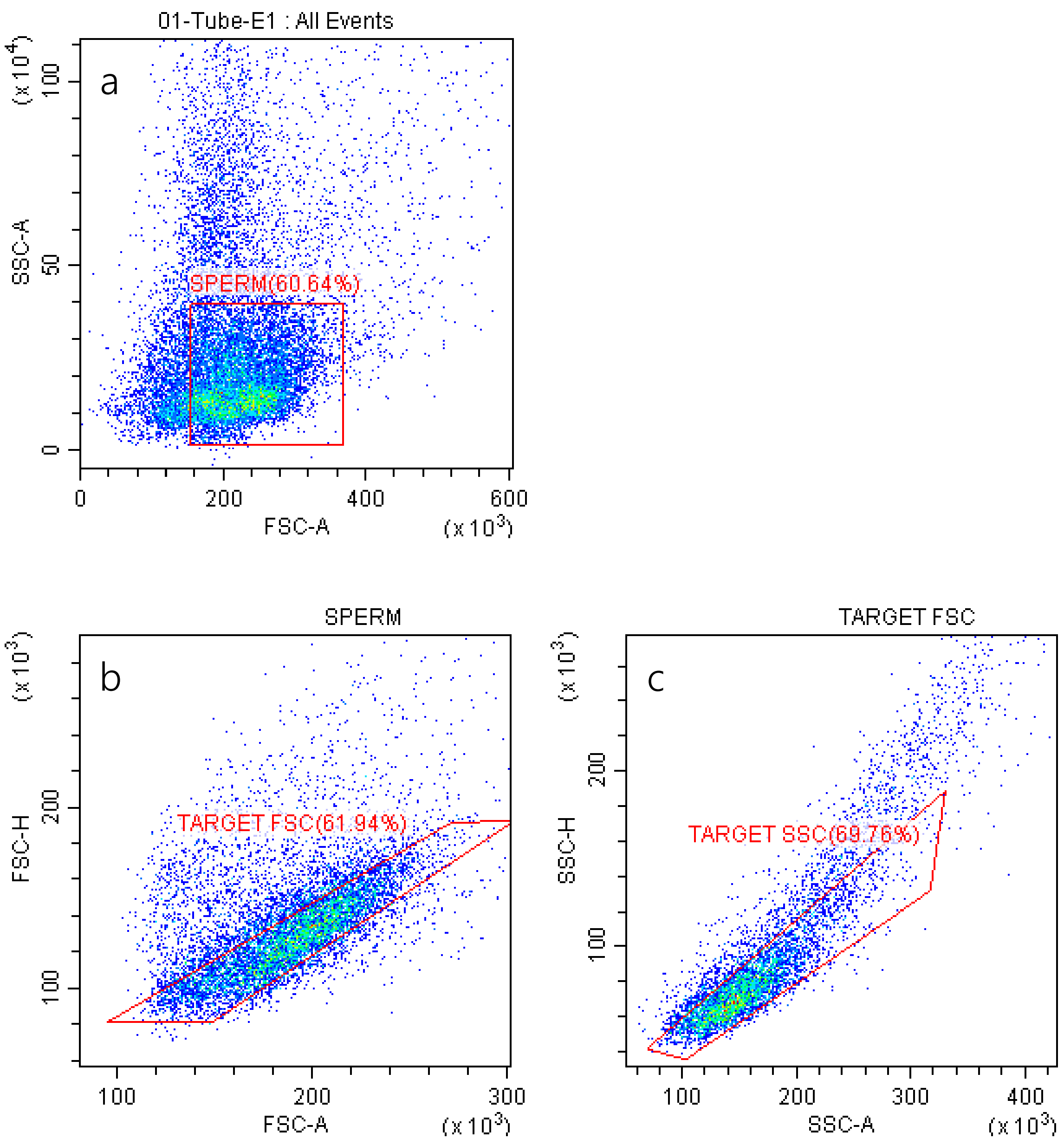

Supplemental Figure S1. Graphical represention of a) the gating of sperm events in a contour dot plot of the area (A) of the side (SSC) vs. the forward scatter (FSC) signal, b) the primary gating of singlets in the sperm population (TARGET FSC) by plotting the height (H) vs. the area of the FSC signal, and c) the secondary gating of sperm singlets (TARGET SSC) through an SSC-H vs. SSC-A contour dot plot, for cryopreserved bovine semen doses.
