# Supplementary material for: Sperm Functional Status: A Multiparametric Assessment of the Fertilizing Potential of Bovine Sperm
Source: Vet Sci. 2024 Dec 23;11(12):678. doi: 10.3390/vetsci11120678 (PMC11680172; doi:10.3390/vetsci11120678)

Minimum Information about Flow Cytometric Experiment
(Sperm Chromatin Structure Assay)

1. Experiment Overview

1.1. Purpose

The present study aimed to evaluate sperm DNA integrity, in addition to sperm viability, as predictors of the fertilizing potential of cryopreserved bovine semen after artificial insemination. The integrity of nuclear sperm chromatin structure was tested using the Sperm Chromatin Structure Assay™ (SCSA; Evenson, D., and L. Jost. 2001. Sperm Chromatin Structure Assay for Fertility Assessment. In Current Protocols in Cytometry, S13: 1-27, John Wiley & Sons, Inc., doi 10.1002/0471142956.cy0713s13).

1.2. Keywords

bull; cryopreserved sperm; fertility; artificial insemination; flow cytometry

1.3. Experiment Variables

The percentage of sperm with high DNA fragmentation index (%DFI) was determined in 791 cryopreserved sperm batches.

1.4. Organization

1.4.1. Name: Clinic of Reproductive Medicine, Vetsuisse Faculty, University of Zurich
1.4.2. Address: Winterthurerstrasse 260, CH-8057 Zurich, Switzerland

1.5. Primary Contact

1.5.1. Name: Eleni Malama
1.5.2. Email: emalama@vetclinics.uzh.ch

1.6. Date

Sperm samples were collected and frozen in the years 2012-2022. Flow cytometric analysis was performed within one to three months after the production of samples, in the frame of a regular sperm quality monitoring program.

1.7. Conclusions

The addition of %DFI in the prognostic algorithm that originally included only sperm viability considerably improved the predictability of sperm fertility.

1.8. Quality Control Measures

- A reference sample of cryopreserved bovine sperm (obtained from a proven sperm donor with known sperm quality characteristics) was stained and analyzed in parallel to the experimental samples
- Double aliquots of experimental samples

1.9. Other Relevant Experiment Information

The performance of the SCSA and the analysis of the relevant flow cytometric data followed the protocol guidelines published by Evenson and Jost (2001; Evenson, D., and L. Jost. 2001. Sperm Chromatin Structure Assay for Fertility Assessment. In Current Protocols in Cytometry, S13: 1-27, John Wiley & Sons, Inc., doi 10.1002/0471142956.cy0713s13).

2. Flow Sample / Specimen Details

2.1. Sample / Specimen Material Description

2.1.1. Biological Samples

2.1.1.1. Biological Samples Description: Bovine ejaculates collected in artificial vagina; ejaculates were cryopreserved in liquid nitrogen (-196 °C) after dilution with commercial sperm extender and packaging in 0.25-ml plastic straws

2.1.1.2. Biological sample source description: *Bos taurus taurus*

2.1.1.3. Biological Sample Source Organism Description: Bovine ejaculates collected in artificial vagina; ejaculates were cryopreserved in liquid nitrogen (-196 °C) after dilution with commercial sperm extender (egg yolk-supplemented Triladyl® extender; Minitube, Tiefenbach, Germany) and packaging in 0.25-ml plastic straws

- Taxonomy: *Bos taurus taurus* (breed: Fleckvieh)
- Age: 12 to 122 months old
- Gender: male
- Treatment: N/A
- Other Relevant Biological Sample Source Organism Information:
  All animals were kept in a single artificial insemination center, thus, handled and fed in an identical manner.

2.1.2. Environmental Samples
N/A

2.1.3. Other Samples
N/A

2.2. Sample Characteristics

Expected/analyzed type of cells/particles: spermatozoa, debris

2.3. Sample Treatment Description

- Cryopreserved sperm samples were thawed in waterbath (38 °C, 30 sec)
- Four straws per ejaculate were pooled in single laboratory tube (pre-warmed at 38 °C)
- Sperm samples were diluted to a concentration of 1 to 2 × 10^6 sperm/mL with pre-warmed (38 °C) TNE buffer (0.01 M Tris, 0.15 M NaCl, 1 mM EDTA, pH 7.4) and tested with the SCSA immediately after thawing
- Four hundred (400) µL of acid detergent solution (0.15 M NaCl, 0.08 N HCl, 0.1% Triton-X 100, pH 1.2) were added to 200 µL of diluted semen and thoroughly mixed for 30 sec with a lab vortex; acid detergent solution was kept on ice (approximately 4 °C)
- Then, 1.2 mL of acridine orange (AO) staining solution were added (6.0 µg AO/mL AO staining buffer; AO staining buffer: 0.2 M Na_2HPO_4, 1 mM EDTA, 0.15 M NaCl, 0.1 M citric acid, pH 6.0); AO staining solution was kept on ice (approximately 4 °C)
- Stained samples were flow cytometrically analyzed after exactly 3-minute incubation on ice

2.4. Fluorescence Reagent Description

Each sample has been stained and flow cytometrically assessed according to the following table:

Excitation laser	Blue (488 nm)	Blue (488 nm)
Optical detector	FITC (525/40 BP)	ECD (610/20 BP)
Reporter	Acridine orange (AO)	Acridine orange (AO)
Concentration	6.0 µg AO/mL AO staining buffer	6.0 µg AO/mL AO staining buffer
Manufacturer	Polysciences Inc.	Polysciences Inc.
Cat#	04539	04539
Sample	Sperm	Sperm
Analyte	dsDNA	ssDNA
Characteristic	Integrity of nuclear chromatin structure	Integrity of nuclear chromatin structure

BP, band-pass filter; AO staining buffer, 0.2 M Na_2HPO_4, 1 mM EDTA, 0.15 M NaCl, 0.1 M citric acid, pH 6.0; ds, double-stranded; ss, single-stranded

3. Instrument Details

3.1. Instrument Manufacturer

Beckman Coulter, Inc.
https://www.beckmancoulter.com/

3.2. Instrument Model
CytoFLEX V0-B5-R0 Flow Cytometer (5 detectors, 1 laser)
https://www.beckman.ch/flow-cytometry/research-flow-cytometers/cytoflex/b53018
Technical specification at
https://www.beckmancoulter.com/wsrportal/techdocs?docname=4237298CA

3.3. Instrument Configuration and Settings

3.3.1. Flow cell and fluidics
The instrument has not been altered; alignment-free integrated optics quartz flow cell design (420 µm ×180 µm internal diameter) with >1.3 numerical aperture

3.3.2. Light Sources
- 488-nm, 50-mW solid-state diode laser (blue laser)

3.3.3. Excitation Optics Configuration
The instrument has not been altered. The optical configuration is presented above in the table of *2.4. Fluorescence Reagent Description paragraph.*

3.3.4. Optical Filters
The instrument has not been altered; all filters were original and came with the instrument.

4. Data Analysis Details

4.1. List-mode Data Files
FCS files can be available upon the agreement of the Besamungsverein Neustadt an der Aisch, Germany, after conducting Mathias Siuda (Clinic of Reproductive Medicine, Department for Farm Animals, Vetsuisse Faculty, University of Zurich; msiuda@vetclinics.uzh.ch).

4.2. Compensation Description
N/A

4.3. Data Transformation Details

4.3.1. Purpose of Data Transformation
Graphical illustration and gating

4.3.2. Data Transformation Description
The following visualization settings have been used for gating:
- FSC and SSC: linear scale
- All fluorescence parameters: linear scale

4.3.3. Other Relevant Data Transformation Details

Data analysis was performed using the FCS EXPRESS 4 Flow Cytometry Research Edition (4.07.0005 version) software (De Novo Software, Glendale, U.S.A.)

4.4. Gating (Data Filtering) Details

The same gating strategy has been used for all data files.

4.4.1. Gate Description

The following gates were applied:

- Side scatter area (SSC-A) vs. forward scatter area (FSC-A) gate to define sperm cells (SPERM; Figure 1)
- FSC-height (H) vs FSC-A gate (Figure 2, panel a) and SSC-H vs. SSC-A gate (Figure 2, panel b) to exclude doublets; events of the diagonal population were considered singlets and further used for analysis (TARGET FSC and TARGET SSC, respectively)
- FITC-A (green fluorescence) vs. ECD-A (red fluorescence) gates to define the main diagonal population and the events out of the main population (Figure 3)
- The alpha t (α_t) parameter (also known as DNA fragmentation index, DFI) was computed according to the formula:
  $$\alpha_t = \frac{red\ fluorescence}{total\ fluorescence} \times 1{,}000$$
  where *total fluorescence = red + green fluorescence*
- All sperm fluorescence signals were plotted in an α_t histogram (Figure 4, panel a) and an α_t vs. total fluorescence cytogram (Figure 4, panel b); from the former, the mean α_t value and its SD (i.e. the mean DFI and the SD of DFI of the sperm sample, respectively) were calculated
- The α_t histogram (Figure 4, panel a) was also used for the calculation of %DFI. A region (M1) that started at the right-hand side of the main peak of α_t distribution and stretched to the upper channel was defined. The left-hand boundary of the above-mentioned region was set based on a previously examined reference sperm sample of known α_t distribution characteristics. Events with an α_t value within the M1 region were considered as cells out of the main population with a high DFI value; the percentage of these events in the total population of sperm was quantified as %DFI

4.4.2. Gate Boundaries

Figure 1: *SSC-A vs. FSC-A gate to define sperm*

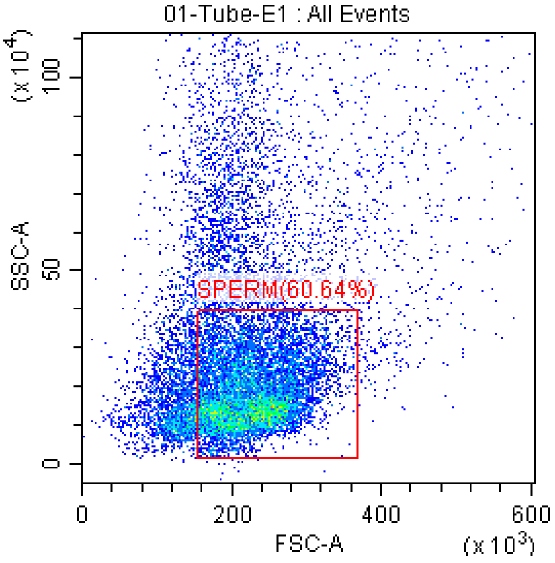

Figure 2: *FSC-H vs. FSC-A gate (panel a) and SSC-H vs. SSC-A gate (panel b) to discriminate doublets. The events of the diagonal gates (TARGET FSC and TARGET SSC, respectively) were considered singlets and further used for analysis.*

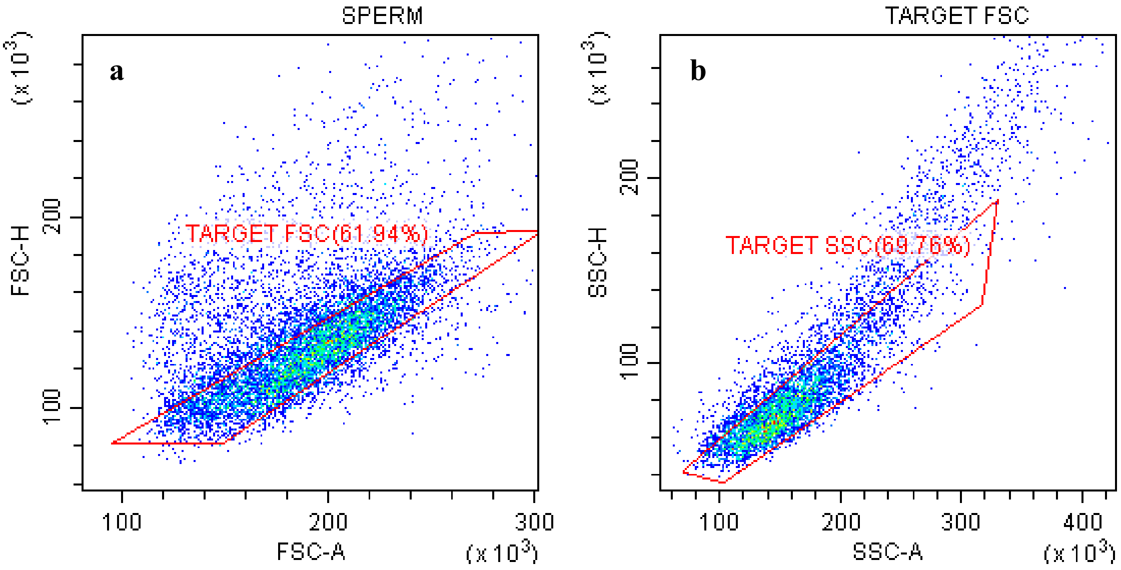

Figure 3: *FITC-A (green fluorescence) vs. ECD-A (red fluorescence) gates to define the main population (GREEN) and the events out of the main population (RED)*

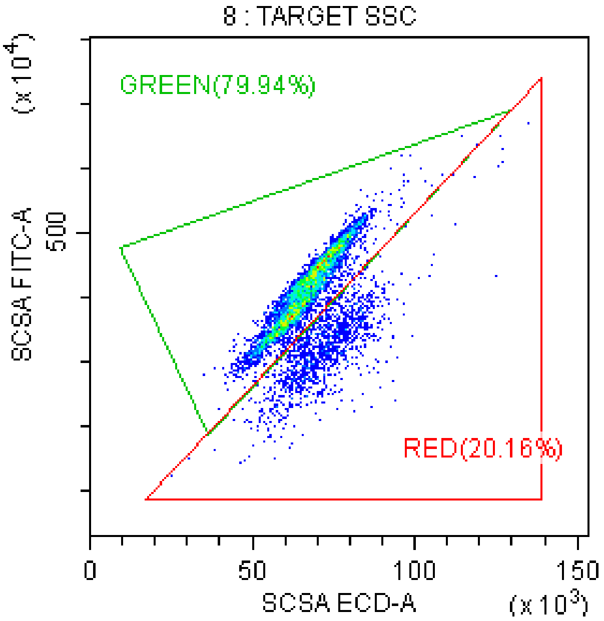

Figure 4: *Sperm fluorescence signals gated in an α_t histogram (panel a) and an α_t vs. total fluorescence cytogram (panel b) for the determination of mean DFI, SD of DFI, and %DFI; red-colored events in the cytogram of panel B indicate the cells out of main population with high DFI (%DFI)*

a

b

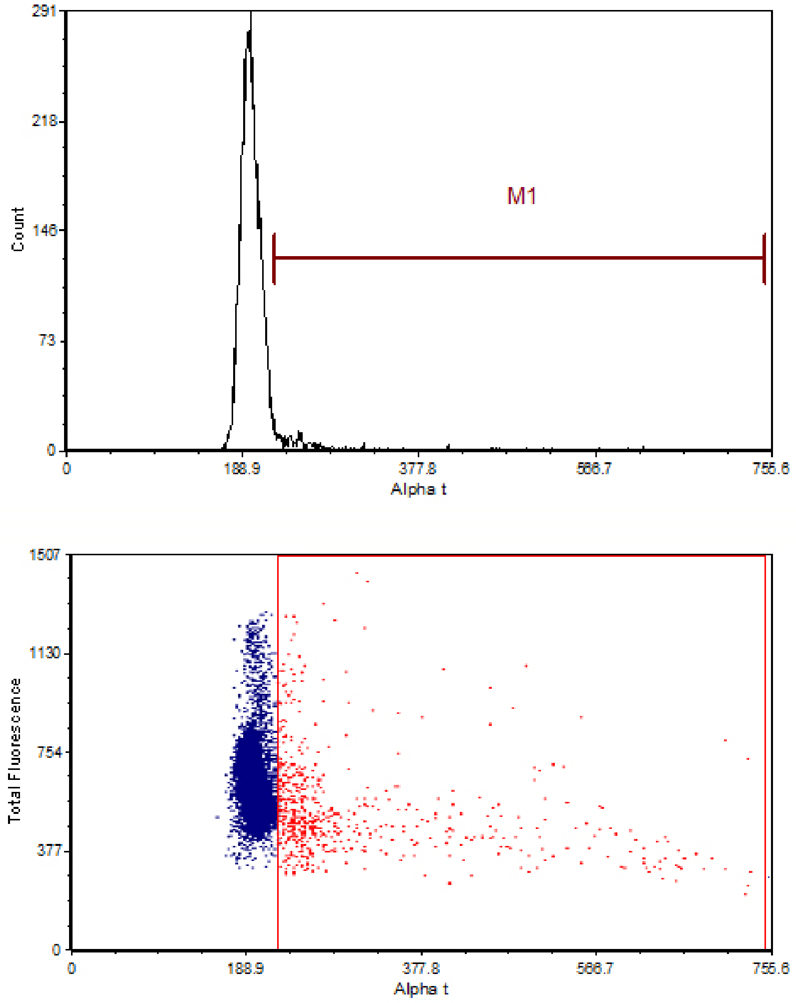

Supplement: Supplementary file 1 [file vetsci-11-00678-s001.zip › Supplemental File S1.pdf]
