# Supplementary material for: Sperm Functional Status: A Multiparametric Assessment of the Fertilizing Potential of Bovine Sperm
Source: Vet Sci. 2024 Dec 23;11(12):678. doi: 10.3390/vetsci11120678 (PMC11680172; doi:10.3390/vetsci11120678)

Minimum Information about Flow Cytometric Experiment
(Five-color assay for sperm quality assessment)

1. Experiment Overview

1.1. Purpose
The present study aimed to evaluate the functional status and the viability of sperm as predictors of the fertilizing potential of cryopreserved bovine semen after artificial insemination.

1.2. Keywords
bull; cryopreserved sperm; fertility; artificial insemination; flow cytometry

1.3. Experiment Variables
The subpopulations of cells with: a) intact plasma membrane and unstained acrosome (viable sperm; PMAI), and b) high esterase activity, intact plasma membrane, unstained acrosome, low intracellular Ca^{2+} levels and high mitochondrial membrane potential ($C_{pos}PI_{neg}PNA_{neg}F_{neg}M_{pos}$ sperm) were quantified as percentages (%) within the total sperm population in 733 cryopreserved semen doses produced from Fleckvieh bulls.

1.4. Organization
1.4.1. Name: Clinic of Reproductive Medicine, Vetsuisse Faculty, University of Zurich
1.4.2. Address: Winterthurerstrasse 260, CH-8057 Zurich, Switzerland

1.5. Primary Contact
1.5.1. Name: Eleni Malama
1.5.2. Email: emalama@vetclinics.uzh.ch

1.6. Date
Sperm samples were collected and cryopreserved in the year 2012-2022. Flow cytometric analysis was performed within one to three months after the production of samples, in the frame of a regular sperm quality monitoring program.

1.7. Conclusions
In this experiment, the majority of sperm examined had ≥40% PMAI and, thus, showed limited variation of their PMAI values. Furthermore, PMAI and $C_{pos}PI_{neg}PNA_{neg}F_{neg}M_{pos}$ values were strongly correlated. The analysis of the data revealed that the assessment of the functional status of sperm ($C_{pos}PI_{neg}PNA_{neg}F_{neg}M_{pos}$ sperm) did not significantly add to the fertility predictive value of a scheme based on sperm viability (PMAI) alone. The combination of sperm viability and functional status were not conclusive about the fertility of bovine cryopreserved batches.

1.8. Quality Control Measures

- Weekly verification of flow cytometer's optical alignment and fluidics system using CytoFLEX Daily QC Fluorospheres (3 µM; https://www.beckman.ch/reagents/coulter-flow-cytometry/qc-and-support-reagents/b53230)
- A reference sample of cryopreserved bovine sperm (obtained from a proven mature sperm donor with known sperm quality characteristics) was stained and analyzed in parallel to the experimental samples

1.9. Other Relevant Experiment Information
N/A

2. Flow Sample / Specimen Details

2.1. Sample / Specimen Material Description

2.1.1. Biological Samples

2.1.1.1. Biological Samples Description: Bovine ejaculates collected in artificial vagina; ejaculates were cryopreserved in liquid nitrogen (-196 °C) after dilution with commercial sperm extender (egg yolk-supplemented Triladyl® extender; Minitube, Tiefenbach, Germany) to a concentration of 60-80×10^6 sperm cells/ml and packaging in 0.25-ml plastic straws.

2.1.1.2. Biological sample source description: *Bos taurus taurus*

2.1.1.3. Biological Sample Source Organism Description: Bovine ejaculate collected in artificial vagina; ejaculates were cryopreserved in liquid nitrogen (-196 °C) after dilution with commercial sperm extender and packaging in 0.25-ml plastic straws

- Taxonomy: *Bos taurus taurus* (breed: Fleckvieh)
- Age: 12 to 116 months old
- Gender: male
- Treatment: N/A
- Other Relevant Biological Sample Source Organism Information:
  All animals were kept in a single artificial insemination center, thus, handled and fed in an identical manner.

2.1.2. Environmental Samples
N/A

2.1.3. Other Samples
N/A

2.2. Sample Characteristics
Expected/analyzed type of cells/particles: bovine spermatozoa, debris

2.3. Sample Treatment Description

- Cryopreserved sperm samples were thawed in a waterbath (38 °C, 30 sec)
- Four straws per cryopreserved sample (ejaculate) were pooled immediately after thawing
- Sperm were diluted to a concentration of 1.2 × 10^6 sperm/mL with pre-warmed (38 °) Tyrode's solution at a final volume of 244.75 µL in a 250-µL reaction well of a 96-well plate
- Staining solution (5.25 mL) was added to diluted sperm in form of a master-mix of the five fluorescent probes with 0.375 µl of calcein violet AM, 1.5 µl of PI working solution, 0.5 µl of PE-PNA, 2.5 µl of Fluo-4 AM and 0.375 µl of $DilC_1(5)$ per reaction well (concentrations of working solutions presented in table of *2.4. Fluorescence Reagent Description paragraph*)
- Stained sperm were incubated at 38 °C in the dark for 15 min
- Samples were flow cytometrically analyzed

2.4. Fluorescence Reagent Description

Each sample has been stained and flow cytometrically analyzed according to the following table:

Excitation laser	Violet (405 nm)	Blue (488 nm)	Blue (488 nm)	Blue (488 nm)	Red (638 nm)
Optical detector	V450 (450/45 BP)	FITC (525/40 BP)	PE (585/42 BP)	PC5.5 (690/50 BP)	APC (660/20 BP)
Reporter	Calcein violet	Fluo-4 AM	PE-PNA	Propidium iodide	$DiIC_1(5)$
Concentration	1.21 µM	2 µM	1 mg/ml	2.99 mM	0.015 µM
Manufacturer	ThermoFischer Scientific	ThermoFischer Scientific	GeneTex	Sigma-Aldrich	ThermoFischer Scientific
Cat#	C34858	F14201	GTX01509	25535-16-4	M34151
Sample	Sperm	Sperm	Sperm	Sperm	Sperm
Analyte	Ubiquitous intracellular esterase	Intracellular Ca^{2+}	Outer acrosomal membrane	Nuclear DNA	Mitochondrial membrane
Characteristic	Intracellular esterase activity (viability)	Intracellular Ca^{2+} levels	Acrosomal status	Plasma membrane integrity (viability)	Mitochondrial membrane potential
Targeted cells	Calcein-positive (C_{pos})	Fluo-4 AM-negative (F_{neg})	PE-PNA-negative (PNA_{neg})	Propidium iodide-negative (PI_{neg})	$DiIC_1(5)$-positive (M_{pos})

BP, band-pass filter; PE-PNA, phycoerythrin-conjugated agglutinin of *Arachis hypogaea*; $DiIC_1(5)$, 1,1',3,3,3',3'-Hexamethylindodicarbocyanine iodide

3. Instrument Details

3.1. Instrument Manufacturer

Beckman Coulter, Inc.

https://www.beckmancoulter.com/

3.2. Instrument Model

CytoFLEX Flow Cytometer V5-B5-R3 (13 detectors, 3 lasers)

https://www.beckman.ch/flow-cytometry/research-flow-cytometers/cytoflex/b53000

Technical specification at http://www.pedsresearch.org/uploads/blog/doc/Cytoflex-Manual.pdf

3.3. Instrument Configuration and Settings

3.3.1. Flow cell and fluidics

The instrument has not been altered; alignment-free integrated optics quartz flow cell design (420 µm ×180 µm internal diameter) with >1.3 numerical aperture

3.3.2. Light Sources

The instrument has not been altered; three-laser configuration

- 405-nm, 80-mW solid-state diode laser (violet laser)
- 488-nm, 50-mW solid-state diode laser (blue laser)
- 638-nm, 50-mW solid-state diode laser (red laser)

3.3.3. Excitation Optics Configuration

The instrument has not been altered. The optical configuration is presented above in the table of *2.4. Fluorescence Reagent Description paragraph.*

3.3.4. Optical Filters

The instrument has not been altered; all filters are original and came with the instrument. The instrument was equipped with a set of wavelength division multiplexers (WDM). Each WDM is a unique detector array that corresponds to a different laser. Each WDM contains optical filters and detectors for detecting channel fluorescence or scatter from a particular laser. The optical configuration is presented above in the table of *2.4. Fluorescence Reagent Description paragraph.*

4. Data Analysis Details

4.1. List-mode Data Files

FCS files can be available upon the agreement of the Besamungsverein Neustadt an der Aisch, Germany, after conducting Mathias Siuda (Clinic of Reproductive Medicine, Department for Farm Animals, Vetsuisse Faculty, University of Zurich; msiuda@vetclinics.uzh.ch).

4.2. Compensation Description

To address the problematic of spectral overlapping a compensation matrix was computed. Compensation was performed post-acquisition according to the following compensation matrix.

Autofluorescence	Channel	-FITC%	-PE%	-PC5.5%	-APC%	-V450%

63.85	FITC		0.50	0.15	0.25	0.00
39.29	PE	84.05		17.25	0.00	0.00
6.23	PC5.5	13.15	0.00		2.75	0.04
4.35	APC	0.00	0.00	0.00		3.87
5.85	V450	0.00	0.00	0.00	0.00	

4.3. Data Transformation Details

4.3.1. Purpose of Data Transformation
Graphical illustration and gating

4.3.2. Data Transformation Description
The default visualization settings of CytExpert Software for CytoFLEX version 2.1 have been used for gating:

- FSC and SSC: linear scale
- All fluorescence parameters: logarithmic scale

4.4. Gating (Data Filtering) Details

The same gating strategy has been used for all data files.

4.4.1. Gate Description
The following gates were applied for the identification of single sperm cells and sperm sub-populations with specific cellular characteristics:

- Side scatter area (SSC-A) vs. forward scatter area (FSC-A) gate to define sperm cells (SPERM; Figure 1)
- FSC-height (H) vs FSC-A gate (Figure 2, panel a) and SSC-H vs. SSC-A gate (Figure 2, panel b) to exclude doublets; events of the diagonal population were considered singlets and further used for analysis (TARGET FSC and TARGET SSC, respectively)
- For the determination of PMAI sperm: PE vs. PC5.5 gate to define $PI_{neg}PNA_{neg}$ (PMAI) sperm (Figure 3)
- For the determination of $C_{pos}PI_{neg}PNA_{neg}F_{neg}M_{pos}$ sperm: APC vs. V450 gate to define $C_{pos}M_{pos}$ sperm (Figure 4); PC5.5 vs. FITC gate applied to $C_{pos}M_{pos}$ sperm to define the $PI_{neg}F_{neg}$ sperm, i.e., the $C_{pos}PI_{neg}F_{neg}M_{pos}$ sperm in the total population (Figure 5); PE gate (histogram) applied to $C_{pos}PI_{neg}F_{neg}M_{pos}$ sperm to define the PNA_{neg} subpopulation and finally the $C_{pos}PI_{neg}PNA_{neg}F_{neg}M_{pos}$ sperm in that total population. The gating hierarch is graphically summarized in Figure 6

The positive as well as the negative sperm sub-populations were also checked through fluorescence histogram for single colors.

4.4.2. Gate Boundaries

Figure 1: *SSC-A vs. FSC-A gate to define sperm*

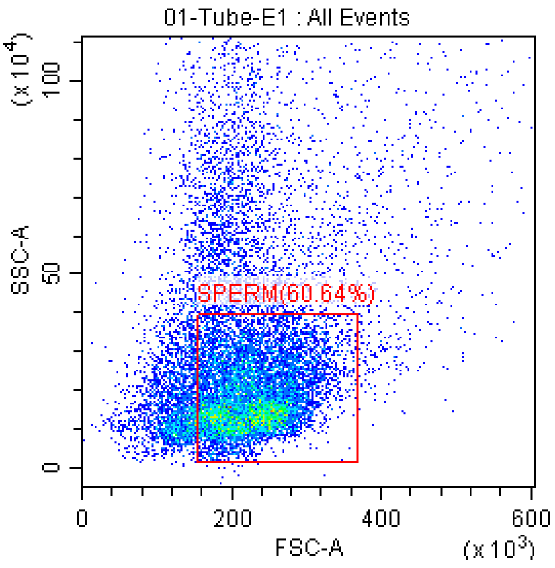

Figure 2: *FSC-H vs. FSC-A gate (panel A) and SSC-H vs. SSC-A gate (panel B) to discriminate doublets. The events of the diagonal gates (TARGET FSC and TARGET SSC, respectively) were considered singlets and further used for analysis.*

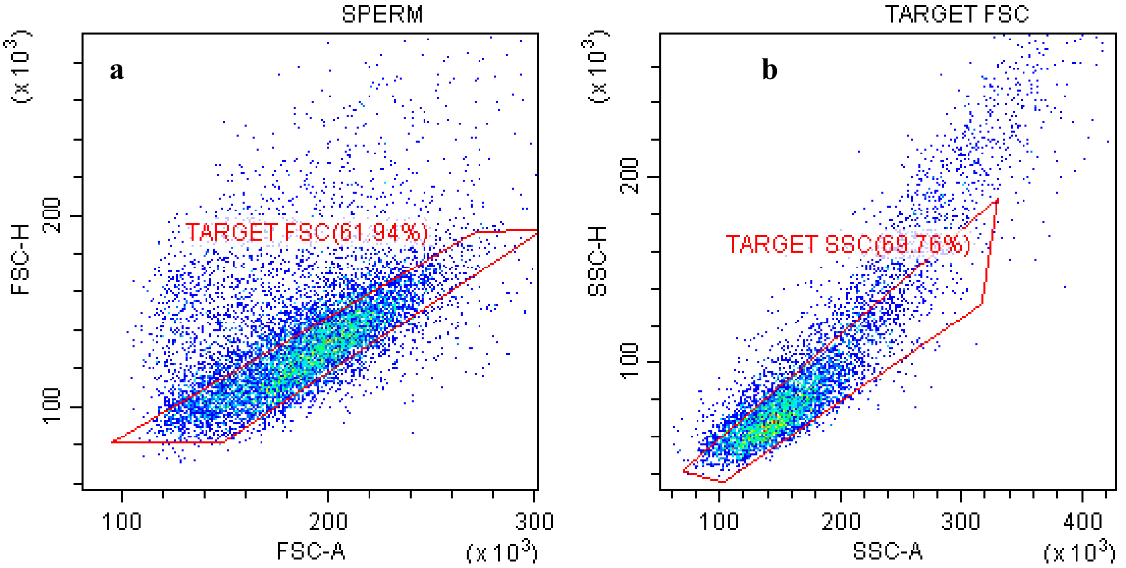

Figure 3:

PC5.5 vs. PE gate to define $PI_{neg}PNA_{neg}$ sperm (PMAI; events of the lower-left quadrant)

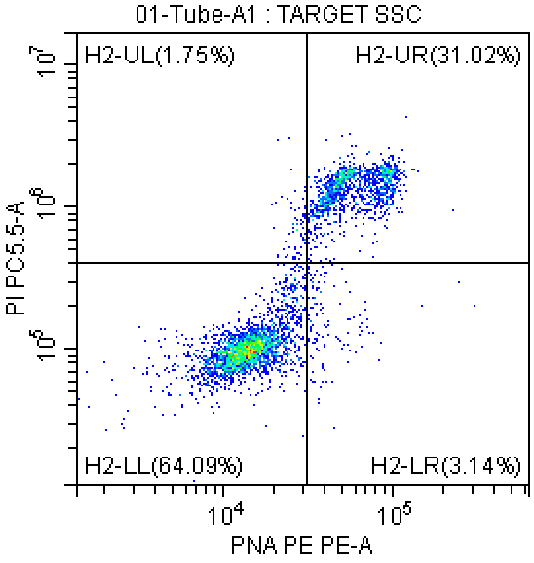

Figure 4:

APC vs. V450 gate to define $C_{pos}M_{pos}$ sperm (events of the upper-right quadrant)

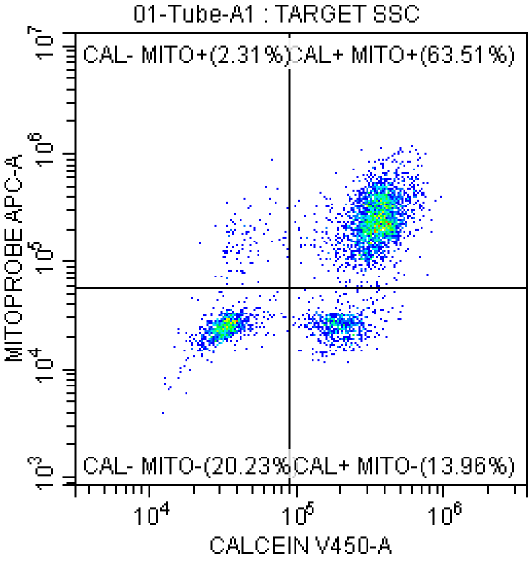

Figure 5:

PC5.5 vs. FITC gate to define $PI_{neg}F_{neg}$ sperm

(events of the lower-left gate)

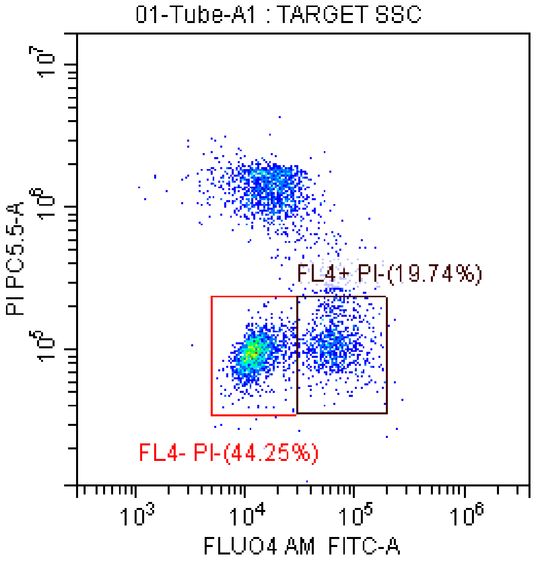

Figure 6. Overview of the gating hierarchy applied for the determination of $C_{pos}PI_{neg}PNA_{neg}F_{neg}M_{pos}$ sperm; APC vs. V450 gate to define $C_{pos}M_{pos}$ sperm (stage I); PC5.5 vs. FITC gate applied to $C_{pos}M_{pos}$ sperm to define the $PI_{neg}F_{neg}$ sperm (stage II); PE gate (histogram) applied to $C_{pos}PI_{neg}F_{neg}M_{pos}$ to define the PNA_{neg} sperm

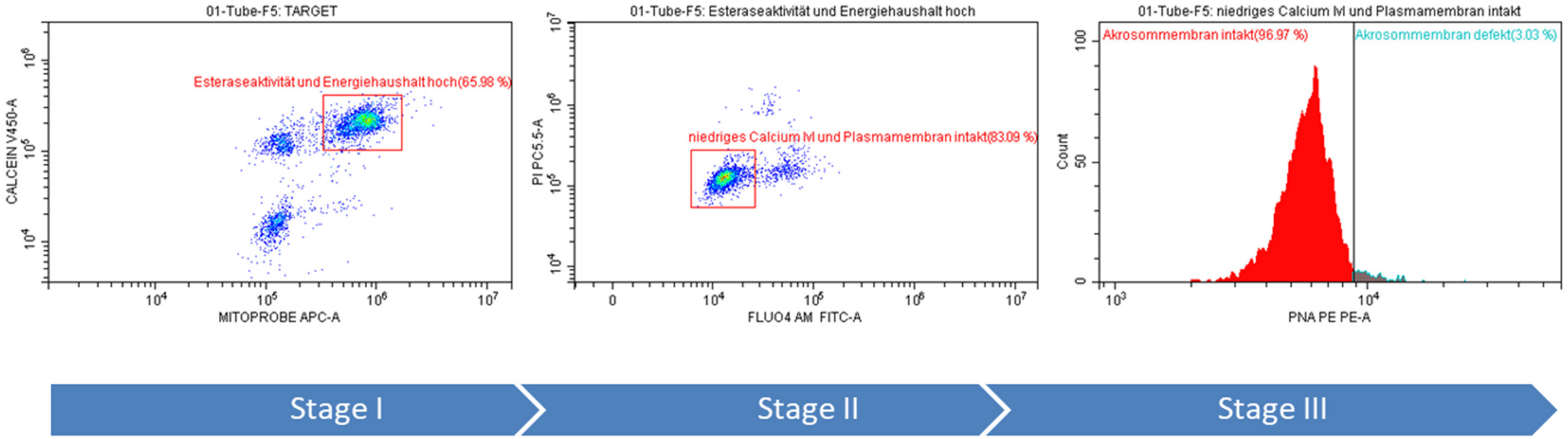

Supplement: Supplementary file 1 [file vetsci-11-00678-s001.zip › Supplemental File S2.pdf]
