# Supplementary material for: Sperm Functional Status: A Multiparametric Assessment of the Fertilizing Potential of Bovine Sperm
Source: Vet Sci. 2024 Dec 23;11(12):678. doi: 10.3390/vetsci11120678 (PMC11680172; doi:10.3390/vetsci11120678)
Supplement: Supplementary file 1 [file vetsci-11-00678-s001.zip › Supplemental Table S1.pdf]

Supplemental Table S1. Descriptive statistics (number of records N, mean ± SD) for the percentage of sperm with an intact plasma membrane (PMAI), the percentage of sperm with high esterase activity, intact plasma membrane and acrosome, low intracellular Ca^{2+} levels and high mitochondrial membrane potential ($C_{pos}PI_{neg}PNA_{neg}F_{neg}M_{pos}$), and the percentage of sperm with a high DNA fragmentation index (%DFI) in cryopreserved bovine sperm batches, in condition to the age class of the bull on the day of batch production. The *p* values computed after performing the Kruskal–Wallis rank sum test for age–class-related differences in the variance of sperm traits are presented; significant age–class-related differences within a row are flagged with different superscript letters.

Sperm characteristic	Overall		Young (<24 months)		Mature (24-84 months)		Old (>84 months)		P value
	N	Mean ± SD	N	Mean ± SD	N	Mean ± SD	N	Mean ± SD	
PMAI sperm (%)	10'427	56.27 ± 9.93	1'696	56.07 ± 9.81[a]	6'201	56.81 ± 9.82[b]	2'530	55.07 ± 10.17[c]	<0.001
$C_{pos}PI_{neg}PNA_{neg}F_{neg}M_{pos}$ sperm (%)	2'217	42.54 ± 9.60	980	43.06 ± 8.94[a]	1'116	42.37 ± 10.18[a]	121	39.91 ± 8.77[b]	0.001
%DFI (%)	3'165	4.63 ± 3.01	1'390	5.10 ± 3.56[a]	1'368	4.07 ± 2.12[b]	407	4.92 ± 3.22[a]	<0.001
