# Supplementary material for: Sperm Functional Status: A Multiparametric Assessment of the Fertilizing Potential of Bovine Sperm
Source: Vet Sci. 2024 Dec 23;11(12):678. doi: 10.3390/vetsci11120678 (PMC11680172; doi:10.3390/vetsci11120678)
Supplement: Supplementary file 1 [file vetsci-11-00678-s001.zip › Supplemental Table S2.pdf]

Supplemental Table S2. Descriptive statistics (number of records N, mean ± SD for continuous variable, and absolute and percentual number of observations for the categorical variables) for the number of first services, the batch-specific non-return rate (NRR) within 60–90 days after artificial insemination, the age of sire on the day of batch production and the bull breed, in condition to the age class of the bull on the day of cryopreserved sperm batch production. The *p* values computed after performing the Kruskal–Wallis rank sum test for age–class-related differences in the number of first services and the NRR are presented; significant age–class-related differences within a row are flagged with different superscript letters.

Characteristic	Overall, N = 24'433	Young (<24 months), N = 13'143	Mature (24-84 months), N = 8'144	Old (>84 months), N = 3'147	P value
Number of first services	89.21 ± 101.88	70.69 ± 73.34[a]	109.08 ± 121.70[b]	115.14 ± 129.12[c]	<0.001
60 to 90-day NRR (%)	63.53 ± 22.50	62.97 ± 22.14[a]	63.98 ± 23.19[a]	64.72 ± 22.17[a]	<0.001
Age (months)	40.70 ± 31.12	16.40 ± 2.18	60.79 ± 17.85	97.36 ± 12.15	
Breed					
Aberdeen Angus	99 / 26,220 (0.4%)	71 / 14,465 (0.5%)	28 / 8,605 (0.3%)	0 / 3,150 (0%)	
Blonde d' Aquitain	1 / 26,220 (<0.1%)	0 / 14,465 (0%)	0 / 8,605 (0%)	1 / 3,150 (<0.1%)	
Brown Swiss	3 / 26,220 (<0.1%)	1 / 14,465 (<0.1%)	2 / 8,605 (<0.1%)	0 / 3,150 (0%)	
Charolais	22 / 26,220 (<0.1%)	18 / 14,465 (0.1%)	4 / 8,605 (<0.1%)	0 / 3,150 (0%)	
Fleckvieh	25,396 / 26,220 (97%)	13,727 / 14,465 (95%)	8,527 / 8,605 (99%)	3,142 / 3,150 (100%)	
Fleckvieh-meat production direction	66 / 26,220 (0.3%)	57 / 14,465 (0.4%)	9 / 8,605 (0.1%)	0 / 3,150 (0%)	
Gelbvieh	261 / 26,220 (1.0%)	252 / 14,465 (1.7%)	9 / 8,605 (0.1%)	0 / 3,150 (0%)	
Limousin	66 / 26,220 (0.3%)	59 / 14,465 (0.4%)	7 / 8,605 (<0.1%)	0 / 3,150 (0%)	
Pinzgauer	8 / 26,220 (<0.1%)	8 / 14,465 (<0.1%)	0 / 8,605 (0%)	0 / 3,150 (0%)	
Holstein red	15 / 26,220 (<0.1%)	12 / 14,465 (<0.1%)	2 / 8,605 (<0.1%)	1 / 3,150 (<0.1%)	
Holstein-Friesian	257 / 26,220 (1.0%)	236 / 14,465 (1.6%)	16 / 8,605 (0.2%)	5 / 3,150 (0.2%)	
Other breeds	7 / 26,220 (<0.1%)	7 / 14,465 (<0.1%)	0 / 8,605 (0%)	0 / 3,150 (0%)	
Wagyu	17 / 26,220 (<0.1%)	17 / 14,465 (0.1%)	0 / 8,605 (0%)	0 / 3,150 (0%)	
Belgian white-blue	2 / 26,220 (<0.1%)	0 / 14,465 (0%)	1 / 8,605 (<0.1%)	1 / 3,150 (<0.1%)	
