# Supplementary material for: Sperm Functional Status: A Multiparametric Assessment of the Fertilizing Potential of Bovine Sperm
Source: Vet Sci. 2024 Dec 23;11(12):678. doi: 10.3390/vetsci11120678 (PMC11680172; doi:10.3390/vetsci11120678)
Supplement: Supplementary file 1 [file vetsci-11-00678-s001.zip › Supplemental Table S3.pdf]

Supplemental Table S3. Descriptive statistics (number of records N, mean ± SD) for the percentage of sperm with an intact plasma membrane (PMAI), the percentage of sperm with high DNA fragmentation index (%DFI), the number of first services and the non-return rate (NRR) 60–90 days after ≥100 first services for 791 cryopreserved bovine sperm batches (dataset A), in condition to the age class of the bull on the day of batch production. The *p* values computed after performing the Kruskal–Wallis rank sum test for age–class-related differences in the variance of the examined variables are presented; significant age–class-related differences within a row are flagged with different superscript letters.

Characteristic	Overall, N = 791	Young (<24 months), N = 189	Mature (24-84 months), N = 456	Old (>84 months), N = 146	P value
PMAI sperm (%)	56.35 ± 9.87	55.00 ± 9.13^{a}	57.26 ± 9.86^{b}	55.28 ± 10.53^{ab}	0.002
%DFI (%)	4.53 ± 2.38	5.10 ± 2.74^{a}	4.12 ± 1.43^{b}	5.05 ± 3.66^{a}	0.002
Number of first services	219.61 ± 89.64	162.19 ± 52.70^{a}	235.40 ± 88.61^{b}	244.64 ± 99.12^{b}	<0.001
NRR (%)	62.02 ± 5.40	60.31 ± 6.20^{a}	62.45 ± 4.97^{b}	62.87 ± 5.14^{b}	<0.001
