# Supplementary material for: Sperm Functional Status: A Multiparametric Assessment of the Fertilizing Potential of Bovine Sperm
Source: Vet Sci. 2024 Dec 23;11(12):678. doi: 10.3390/vetsci11120678 (PMC11680172; doi:10.3390/vetsci11120678)
Supplement: Supplementary file 1 [file vetsci-11-00678-s001.zip › Supplemental Table S4.pdf]

Supplemental Table S4. Descriptive statistics (number of records N, mean ± SD) for the percentage of sperm with an intact plasma membrane (PMAI) and the percentage of sperm with high esterase activity, intact plasma membrane and acrosome, low intracellular Ca^{2+} levels and high mitochondrial membrane potential ($C_{pos}PI_{neg}PNA_{neg}F_{neg}M_{pos}$), the number of first services and the non-return rate (NRR) 60–90 days after ≥100 first services for 733 cryopreserved bovine sperm batches (dataset B), in condition to the age class of the bull on the day of batch production. The *p* values computed after performing the Kruskal–Wallis rank sum test for age–class-related differences in the variance of the examined variables are presented; significant age–class-related differences within a row are flagged with different superscript letters.

Characteristic	Overall, N = 733	Young (<24 months), N = 241	Mature (24-84 months), N = 433	Old (>84 months), N = 49	P value
PMAI sperm (%)	54.21 ± 10.27	53.26 ± 10.50[a]	55.29 ± 10.09[b]	49.10 ± 8.80[c]	<0.001
$C_{pos}PI_{neg}PNA_{neg}F_{neg}M_{pos}$ sperm (%)	42.23 ± 9.91	42.57 ± 9.39[a]	42.49 ± 10.26[a]	38.13 ± 8.21[b]	0.005
Number of first services	186.11 ± 74.84	147.57 ± 41.65[a]	204.49 ± 78.66[b]	209.45 ± 92.98[b]	<0.001
NRR (%)	62.88 ± 5.33	61.81 ± 6.06[a]	63.51 ± 4.88[b]	62.53 ± 4.65[ab]	<0.001
