# Supplementary material for: Sperm Functional Status: A Multiparametric Assessment of the Fertilizing Potential of Bovine Sperm
Source: Vet Sci. 2024 Dec 23;11(12):678. doi: 10.3390/vetsci11120678 (PMC11680172; doi:10.3390/vetsci11120678)
Supplement: Supplementary file 1 [file vetsci-11-00678-s001.zip › Supplemental Table S5.pdf]

Supplemental Table S5. Pearson's correlation coefficients for the pairwise correlations between the percentage of sperm with an intact plasma membrane (PMAI), the percentage of sperm with a high DNA fragmentation index (%DFI), and the percentage of sperm with high esterase activity, an intact plasma membrane and acrosome, low intracellular Ca^{2+} levels, and high mitochondrial membrane potential ($C_{pos}PI_{neg}PNA_{neg}F_{neg}M_{pos}$) that were flow cytometrically evaluated in a total of 10,427 cryopreserved bovine sperm samples. The PMAI score was assessed by means of dual staining with propidium iodide (PI) and the fluorescein isothiocyanate (FITC)-conjugated peanut agglutinin (PNA) or as part of a five-color staining panel including PI and PNA. The *p* values (non-adjusted and adjusted for multiple tests) and the sample size of each pairwise correlation are also demonstrated.

	PMAI (dual PI/FITC-PNA stain)	%DFI	PMAI (five-colour assay)	$C_{pos}PI_{neg}PNA_{neg}F_{neg}M_{pos}$ sperm
	Correlation matrix (correlation coefficients)			
PMAI (dual PI/FITC-PNA stain)	1.00	-0.16	1.00	0.92
%DFI	-0.16	1.00	-0.28	-0.17
PMAI (five-colour assay)	1.00	-0.28	1.00	0.92
$C_{pos}PI_{neg}PNA_{neg}F_{neg}M_{pos}$ sperm	0.92	-0.17	0.92	1.00
	P values (entries above the diagonal are adjusted for multiple tests)			
PMAI (dual PI/FITC-PNA stain)		<0.001	<0.001	<0.001
%DFI	<0.001		<0.001	<0.001
PMAI (five-colour assay)	<0.001	<0.001		<0.001
$C_{pos}PI_{neg}PNA_{neg}F_{neg}M_{pos}$ sperm	<0.001	<0.001	<0.001	
	Sample size for each pairwise correlation test			
PMAI (dual PI/FITC-PNA stain)	10390	2184	1911	2180
%DFI	2184	3165	641	732
PMAI (five-colour assay)	1911	641	1948	1944
$C_{pos}PI_{neg}PNA_{neg}F_{neg}M_{pos}$ sperm	2180	732	1944	2217
