# Supplementary material for: Sperm Functional Status: A Multiparametric Assessment of the Fertilizing Potential of Bovine Sperm
Source: Vet Sci. 2024 Dec 23;11(12):678. doi: 10.3390/vetsci11120678 (PMC11680172; doi:10.3390/vetsci11120678)
Supplement: Supplementary file 1 [file vetsci-11-00678-s001.zip › Supplemental Table S6.pdf]

Supplemental Table S6. Pearson's correlation coefficients (and respective *p* values) for the pairwise correlations between (a) the percentage of sperm with an intact plasma membrane (PMAI) and the percentage of sperm with a high DNA fragmentation index (%DFI) (dataset A), and (b) the PMAI score and the percentage of sperm with high esterase activity, an intact plasma membrane and acrosome, low intracellular Ca^{2+} levels, and high mitochondrial membrane potential ($C_{pos}PI_{neg}PNA_{neg}F_{neg}M_{pos}$) that were flow cytometrically evaluated in 791 and 733 cryopreserved bovine sperm samples for datasets A and B, respectively. In dataset A, the PMAI score was assessed by means of dual staining with propidium iodide (PI) and the fluorescein isothiocyanate (FITC)-conjugated peanut agglutinin (PNA); the PMAI score was evaluated as part of a five-color staining panel including PI and PNA for dataset B.

Dataset A	Correlation coefficients		P values	
	PMAI (dual PI/FITC-PNA stain)	%DFI	PMAI (dual PI/FITC-PNA stain)	%DFI
PMAI (dual PI/FITC-PNA stain)	1.00	-0.17		<0.001
%DFI	-0.17	1.00	<0.001	

Dataset B	Correlation coefficients		P values	
	PMAI (five-colour assay)	$C_{pos}PI_{neg}PNA_{neg}F_{neg}M_{pos}$ sperm	PMAI (five-colour assay)	$C_{pos}PI_{neg}PNA_{neg}F_{neg}M_{pos}$ sperm
PMAI (five-colour assay)	1.00	0.93		<0.001
$C_{pos}PI_{neg}PNA_{neg}F_{neg}M_{pos}$ sperm	0.93	1.00	<0.001	
