# Supplementary figures and images for: Sperm Functional Status: A Multiparametric Assessment of the Fertilizing Potential of Bovine Sperm
Source: Vet Sci. 2024 Dec 23;11(12):678. doi: 10.3390/vetsci11120678 (PMC11680172; doi:10.3390/vetsci11120678)

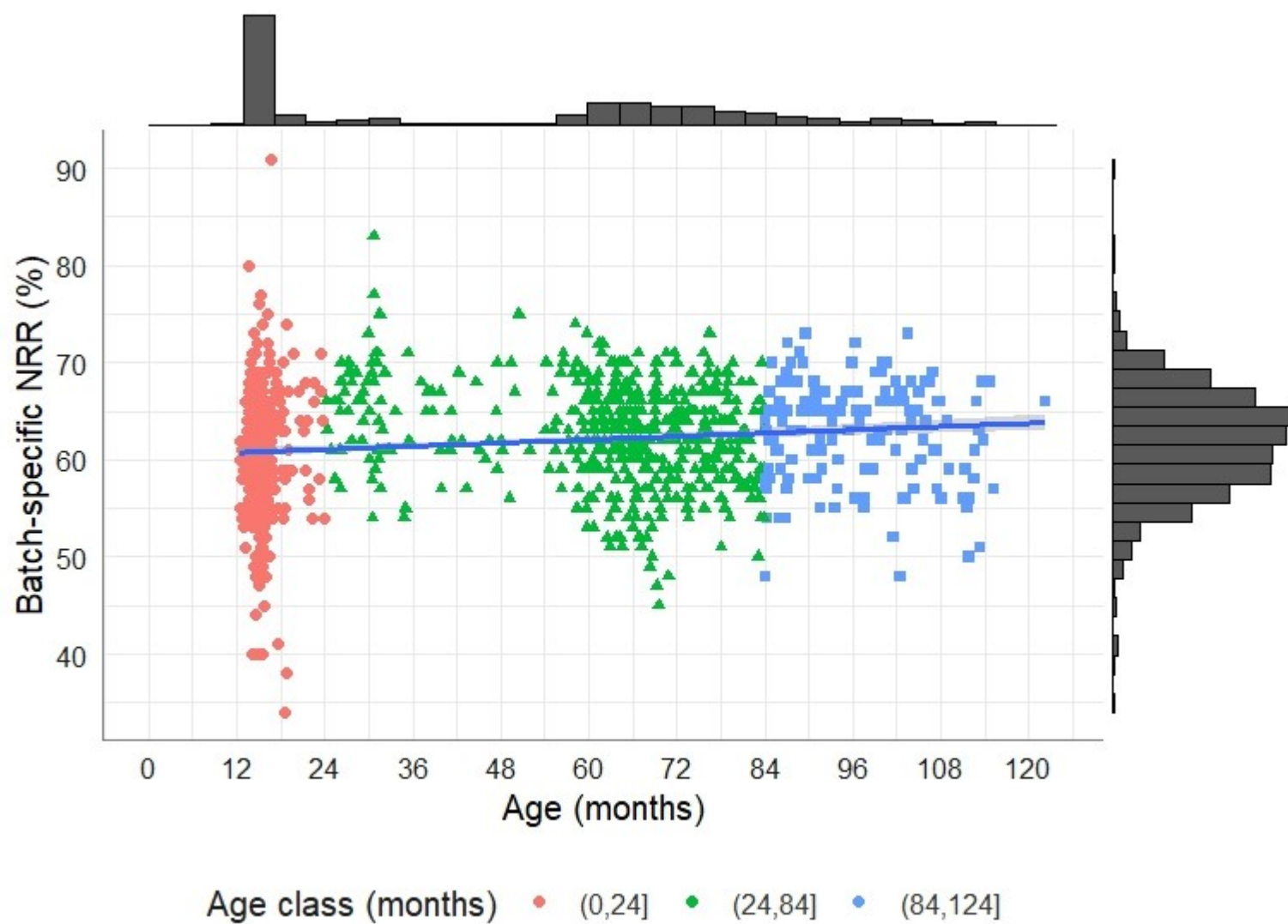

Supplement: Supplementary file 1 [file vetsci-11-00678-s001.zip › Supplemental Figure S2.pdf]
